# Non-Invasive Imaging and Scoring of Peritoneal Metastases in Small Preclinical Animal Models Using Ultrasound: A Preliminary Trial

**DOI:** 10.3390/biomedicines10071610

**Published:** 2022-07-06

**Authors:** Roxan F. C. P. A. Helderman, Mauricio Tobón Restrepo, Hans M. Rodermond, Gregor G. W. van Bochove, Daan R. Löke, Nicolaas A. P. Franken, H. Petra Kok, Pieter J. Tanis, Johannes Crezee, Arlene L. Oei

**Affiliations:** 1 Department of Radiation Oncology, Amsterdam UMC Location University of Amsterdam, Meibergdreef 9, 1105 AZ Amsterdam, The Netherlands; f.c.helderman@amsterdamumc.nl (R.F.C.P.A.H.); h.rodermond@amsterdamumc.nl (H.M.R.); g.g.vanbochove@amsterdamumc.nl (G.G.W.v.B.); d.r.loke@amsterdamumc.nl (D.R.L.); n.a.franken@amsterdamumc.nl (N.A.P.F.); h.p.kok@amsterdamumc.nl (H.P.K.); h.crezee@amsterdamumc.nl (J.C.); 2 Center for Experimental and Molecular Medicine (CEMM), Laboratory for Experimental Oncology and Radiobiology (LEXOR), 1105 AZ Amsterdam, The Netherlands; 3 Cancer Center Amsterdam, Cancer Biology and Immunology, 1105 AZ Amsterdam, The Netherlands; 4 Division of Diagnostic Imaging, Department of Clinical Sciences of Companion Animals, Faculty of Veterinary Medicine, Utrecht University, 3584 CL Utrecht, The Netherlands; m.tobonrestrepo@uu.nl; 5 Department of Surgery, Amsterdam UMC Location University of Amsterdam, Meibergdreef 9, 1105 AZ Amsterdam, The Netherlands; p.j.tanis@amsterdamumc.nl; 6 Department of Surgical Oncology and Gastrointestinal Surgery, Erasmus MC Cancer Institute, 3015 GD Rotterdam, The Netherlands

**Keywords:** peritoneal carcinomatosis, imaging modality, orthotopic animal model, peritoneal cancer index

## Abstract

Background: The peritoneum is a common site for the formation of metastases originating from several gastrointestinal and gynecological malignancies. A representative preclinical model to thoroughly explore the pathophysiological mechanisms and to study new treatment strategies is important. A major challenge for such models is defining and quantifying the (total) tumor burden in the peritoneal cavity prior to treatment, since it is preferable to use non-invasive methods. We evaluated ultrasound as a simple and easy-to-handle imaging method for this purpose. Methods: Peritoneal metastases were established in six WAG/Rij rats through i.p. injections of the colon carcinoma cell line CC-531. Using ultrasound, the location, number and size of intraperitoneal tumor nodules were determined by two independent observers. Tumor outgrowth was followed using ultrasound until the peritoneal cancer index (PCI) was ≥8. Interobserver variability and ex vivo correlation were assessed. Results: Visible peritoneal tumor nodules were formed in six WAG/Rij rats within 2–4 weeks after cell injection. In most animals, tumor nodules reached a size of 4–6 mm within 3–4 weeks, with total PCI scores ranging from 10–20. The predicted PCI scores using ultrasound ranged from 11–19 and from 8–18, for observer 1 and 2, respectively, which was quite similar to the ex vivo scores. Conclusions: Ultrasound is a reliable non-invasive method to detect intraperitoneal tumor nodules and quantify tumor outgrowth in a rat model.

## 1. Introduction

Several gastrointestinal and gynecological malignancies can metastasize to the peritoneum. The presence of peritoneal metastases (PM) is generally associated with poor prognosis and rapid disease progression [1]. The average survival of patients with PM of colorectal cancer origin ranges between 5–24 months [2,3]. Patients with PM that originate from gastric cancer have a median overall survival of 4–8 months, and a 5-year survival rate of approximately 3–6% [4,5]. Ovarian cancer patients with PM have a 5-year survival rate of 25–29%, compared to a 5-year survival rate of >90% in early-stage ovarian cancer [6]. Besides the more common gastrointestinal and gynecological malignancies, pseudomyxoma peritonei (PMP) and malignant peritoneal mesothelioma (MPM) are more rare peritoneal surface malignancies [7,8,9].

The treatment options for patients with PM are systemic therapy and locoregional treatment modalities. The penetration of systemic chemotherapy into peritoneal lesions is relatively poor, and therefore intraperitoneal chemotherapy has been introduced to increase effectiveness and reduce toxicity. The only treatment with curative intent is cytoreductive surgery (CRS), during which all macroscopic visible tumor lesions are removed, followed by hyperthermic intraperitoneal chemotherapy (HIPEC) to eradicate remaining microscopic disease to prevent recurrence [10]. During HIPEC, a heated chemotherapy solution is circulated in the peritoneal cavity for 30 min up to 120 min. Although HIPEC is a successful treatment, present protocols vary widely and are largely determined by institutional preferences [11].

Representative PM preclinical models are essential to thoroughly explore pathophysiological mechanisms, to study new treatment strategies, and to improve the effectiveness of existing locoregional treatments, e.g., for HIPEC [12]. Orthotopic animal models are increasingly used to study the tumor response in a preclinical research model which mimics the clinical disease process and tumor microenvironment [13]. In small animals, such as mice, rats and hamsters, PM can be induced by the injection of cancer cells into the abdominal cavity. This results in the formation of small tumor lesions throughout the peritoneal cavity, comparable to PM in patients [13,14].

In most studies, a fixed time point is used between the injection of cells and performing experiments. Choosing a fixed time point for therapy does not mean that tumor sizes are similar at the time of treatment. To compensate for the resulting potential heterogeneity in tumor size, a higher number of animals is required, as we need to exclude animals in which tumor sizes are not within the desired treatment range. Accurate non-invasive treatment measurements to study tumor outgrowth would be a solution to reduce the number of animals needed, to increase homogeneity between animals regarding tumor sizes, and to improve data reproducibility.

The imaging modalities used for the diagnosis of PM in humans include ultrasound, computed tomography (CT) scans, magnetic resonance imaging (MRI) or positron emission tomography (PET) scans, with abdominopelvic CT scanning as first line detection method [15]. The choice of imaging modality mainly depends on the type of cancer, the extent of the disease, and the disease area. Ultrasound is used in the assessment of abdominal collections and allows for the identification of ascites, but has limitations for the detection of peritoneal nodules [16]. Overall, imaging and diagnosis of PM is challenging and the extent of the disease is often underestimated [17], with reported cases where the detection of PM is an unexpected incidental finding during surgical exploration.

Detecting PM is also challenging in orthotopic animal models. Several imaging methods are available for small animal models [18,19,20]. Bioluminescence imaging is a non-invasive, affordable and quite easy-to-handle imaging modality which is used for the detection of PM in small animal models [21,22]. A major drawback is the limited precision to delineate a tumor or multiple tumors in small animal models. Multiple tumor lesions presenting close to each other, as often occurs in a PM model, result in only a single large positive stain [23,24,25].

As such, it is difficult to define the optimal moment to start treatment, because this depends on a specified extent of disease, which is determined though PCI scoring. PCI is a prognostic factor which plays an important role in patient selection, since it is predictive of the chance of achieving a complete cytoreduction [11,26,27]. The PCI score is defined by the exact size and number of tumor nodules present in the peritoneal cavity.

CT, MRI and PET are standard methods used in the clinic, but they are expensive and require extensive training and practice to ensure appropriate usage, especially for preclinical use. Although ultrasound is not sufficiently accurate to diagnose PM in patients [28], in this study, we investigate whether this non-invasive imaging modality is sufficiently accurate for detecting the size and number of tumor lesions spread throughout the abdominal cavity of small animals. Because the application of ultrasound is non-invasive, imaging can be applied repeatedly without resulting in extra discomfort. First, tumor growth in six rats was assessed over time. Second, the extent of the disease was scored by two independent observers, followed an assessment of the interobserver variability and ex vivo correlation. We provide results and guidelines for ultrasound imaging for the detection and scoring of peritoneal metastases in rats.

## 2. Materials and Methods

### 2.1. Orthotopic PM Animal Model

The outgrowth of peritoneal metastases was examined using ultrasound. Six 7–9-week-old female WAG/Rij rats were obtained from Charles River Laboratories Research models and services, Sulzfeld, Germany. The rats were housed in individually ventilated cages (IVC-cages) (four per cage) with corn cob bedding material under standardized conditions: temperature 21 °C, relative humidity 50–60%, 12 h light/12 h dark and with free access to standard water and food without additions. All animals were acclimatized for 1 week before the start of the experiment. The weight of the rats on the day of sacrifice was 135–160 g. All experiments were approved by the Dutch Central Committee of Animal Experiments, with approval code AVD1180020174184, received on 14 February 2019, and carried out in accordance with the Dutch Animal Welfare Act 1997.

CC-531 cells were injected in the peritoneum of 8–10-week-old WAG/rij rats to induce tumor lesions throughout the abdominal cavity. The CC-531 cell line is a colon carcinoma cell line derived from WAG/Rij rats, which were kindly donated by Prof. Dr. Ignace de Hingh, Catharina Cancer Institute (Eindhoven, The Netherlands) and Roger Lomme, Radboud University (Nijmegen, The Netherlands). These cells were cultured at least two passages after being thawed in RPMI medium (Gibco, Rockville, MD, USA) containing 25 mM HEPES, supplemented with 10% fetal bovine serum (FBS) (Gibco) and 1% penicillin/streptomycin/glutamine (Gibco). The cells were maintained at 37 °C in a humidified atmosphere of 5% CO_2_ in air. The cell line was verified for authenticity and routinely tested for mycoplasma infection before the start of the experiment.

**Figure 1:**
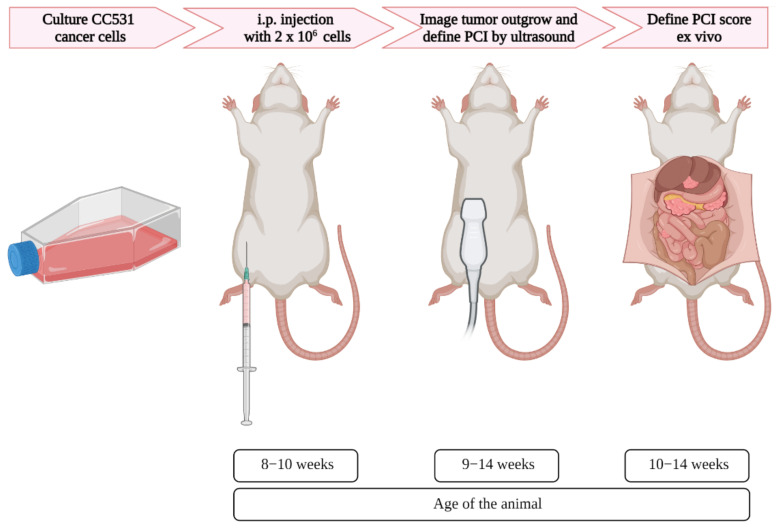
Schematic overview of the induction and imaging of peritoneal metastases in rats, with corresponding age. After the appropriate number of colorectal cancer cells were cultured, the cells were injected in the intraperitoneal area of the rats. Using ultrasound, the tumor formation was assessed, and when tumors reached the size of 4–6 mm, the PCI was scored. Immediately after the last ultrasound imaging, the PCI score was defined ex vivo.

Upon 60–70% confluency, the cells were trypsinized and washed twice with PBS and concentrated to 2 × 10^6^ cells/mL. Subsequently, 1 mL of the cell suspension in culture medium containing 10% FBS was injected intraperitoneally per rat in the right lower part of the abdominal cavity. Ultrasound was used once a week to assess the tumor outgrowth, starting 2 days after cell injection (Figure 1).

### 2.2. Ultrasound Settings for the Detection of Peritoneal Metastases

Ultrasound was performed using the CX50 ultrasound (Philips, Eindhoven, The Netherlands) with probe L15-7io (Philips). The aperture of this probe is 23 mm with 128 elements and it has a nearfield resolution of 1.5–7 mm. This probe is widely applied for vascular ultrasound and for imaging small superficial features, and is therefore suitable for the detection of peritoneal metastases in rats. All images were obtained on the B mode with a frequency of 42 Hz, a focal zone 1–2, a gain of 90, a compress of 66 and a mechanical index of 0.5. The ultrasound parameters with the corresponding settings are presented in Supplementary Figure S1.

### 2.3. Ultrasound Protocol

The ultrasound procedure was performed in a biosafety cabinet. The rats were anesthetized with 0.5–2.5% isoflurane in 100% oxygen using an isoflurane anesthesia vaporizer. Bowel and urinate preparation was not necessary for image tumor outgrowth in this animal model. During preparation, the rats were placed on a heating mat to ensure stable body temperatures. The abdominal skin was shaved and cleaned with 70% ethanol. Ultrasound gel (Aquasonic 100) was applied on the probe and spread over the abdomen. During the ultrasound procedure, it is important to check for abnormalities in the size, shape and location of the organs as well as other remarkable changes.

**Figure 2:**
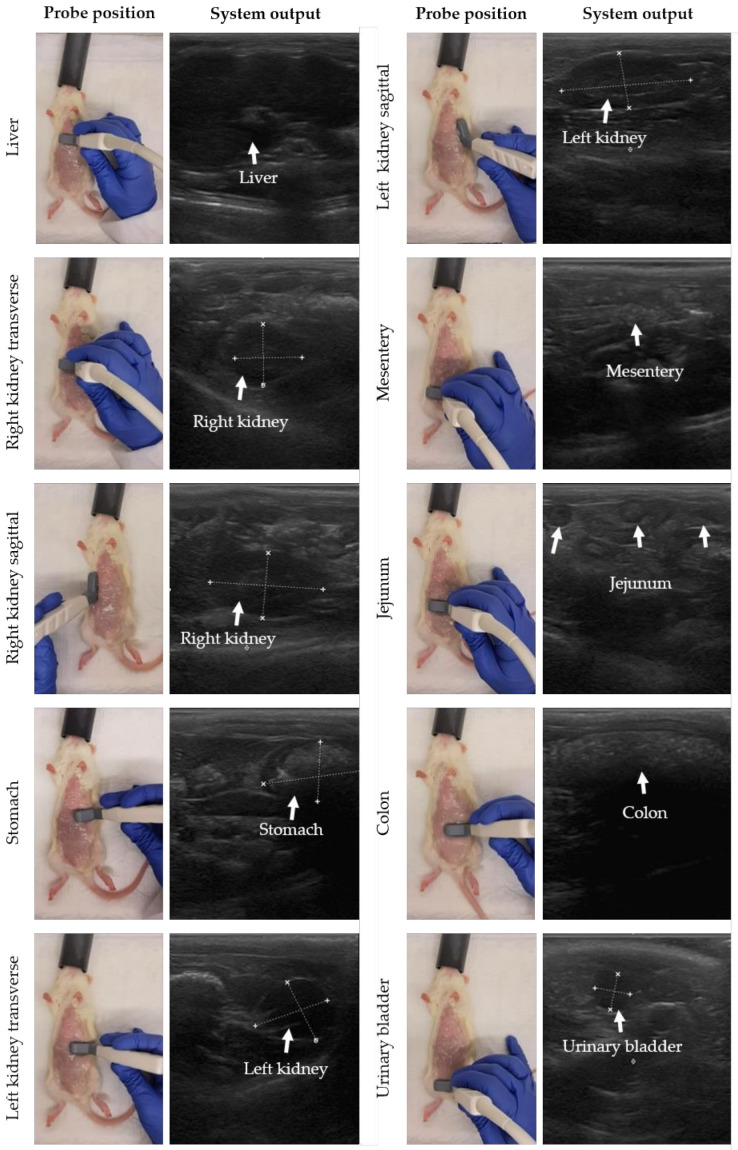
Stepwise movement of probe positions to perform gastrointestinal ultrasound in rats. Before the ultrasound procedure, the abdominal skin was shaved and cleaned with 70% ethanol. For each organ, the probe position with the system output is presented, moving from liver, right kidney, stomach, colon, and left kidney to urinary bladder. In the system output, the organ of interest is highlighted by white measurement marks and/or arrows.

The probe position was moved stepwise to perform gastrointestinal ultrasound in rats. In Figure 2, the probe position with the corresponding system output is presented for each organ following similar descriptions for small animals [29]. The starting point is the detection of the liver. The probe was placed transversally (probe perpendicular to the spine with the indicator to the right of the rat) on the middle of the animal on top of the abdomen and caudal to the sternum. Next, the probe was moved to the right side of the abdomen, and slightly caudal to detect the transverse image of the right kidney. To image the kidney in sagittal, the probe was turned 90 degrees with the indicator towards the head of the rat (probe parallel to the spine). Then, the stomach, spleen and left kidney were detected by shifting the probe transversally to the left cranial and caudal abdomen. Finally, the jejunum, duodenum, colon and urinary bladder were detected in the middle and caudal part of the abdomen and the probe was placed sagittal or transverse to the spine depending on the direction of the intestinal loop. For the urinary bladder, the probe was mainly placed in transverse. Note that the urinary bladder is only visible when filled with urine. Tumor lesions were identified as hypoechoic (dark) rounded structures, sometimes surrounded by a hyperechoic (white) halo, and were mostly superficial. Performing a complete ultrasound procedure required approximately 10–15 min.

### 2.4. Peritoneal Metastasis Scoring System

**Figure 3:**
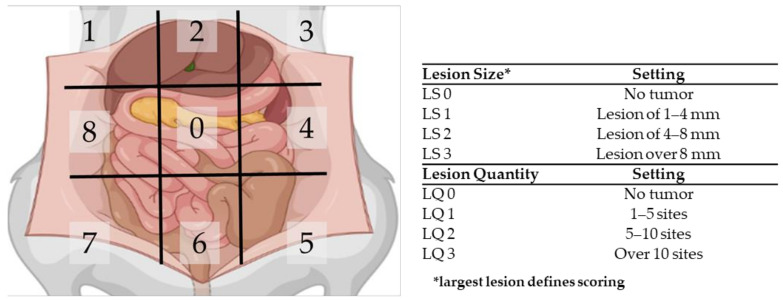
Schematic overview of the peritoneal cancer index (PCI) tool to assess the extent of PM in rats.

Ultrasound was applied weekly on all six rats in order to follow tumor outgrowth. When most tumors reached a size of 4–6 mm, the extent of disease was scored using the PCI. The peritoneal cavity of the rats was divided in nine regions, and for each region, the size and number of lesions was scored. If no tumor nodules could be identified in a region, the local lesion size score (LS) and the lesion quantity score (LQ) were scored as 0. If tumors nodules were present, the size of the biggest nodule was scored with LS1, LS2 or LS3 when the size was 1–4 mm, 4–8 mm or over 8 mm, respectively. The quantity score was given as follows: LQ1 for 1–5 sites, LQ2 for 5–10 sites and LQ3 for over 10 sites per region. In Figure 3, a graphical summary of the PCI scoring system is presented.

### 2.5. PCI Score Prediction

To evaluate whether the ultrasound-based PCI scoring in small animal models is reliable, the PCI score was assessed by two independent observers. When tumor nodules reached a size of 4–6 mm, the observers scored the PCI in vivo using ultrasound, before the animal was sacrificed and PCI was scored ex vivo. Observer 1 (R.F.C.P.A.H.) can be considered as semi-experienced, with 1.5 years of experience using ultrasound on rats only. Observer 2 (A.L.O.) was non-experienced using ultrasound, and only received a short introduction on how to use the ultrasound technique in general and how the application works on rats by observer 1. To reduce the discomfort of the animal, only one observer per ultrasound session scored the PCI. First, the extent of disease was scored on ultrasound by both observers independently. Afterwards, the rats were sacrificed using 100% CO_2_, and the PCI was scored ex vivo before all data were compared.

Observer 1 followed the outgrowth weekly on ultrasound. At the moment tumor nodules reached a size of 4–6 mm, observer 2 scored the PCI on ultrasound once before ex vivo assessment. Within 3 days, PCI was scored on ultrasound by observer 1, and immediately afterwards the animals were sacrificed in order to score the PCI ex vivo. During the scoring procedure, both observers used the ultrasound guidelines provided in this article to cover all regions.

### 2.6. Statistics

The interrater reliability between observer 1 and 2 for scoring the lesion size and quantity on ultrasound were calculated using Cohen’s kappa coefficient.

## 3. Results

In this section, we first show the outgrowth of the injected colon carcinoma cell line in the peritoneal cavity, as determined by ultrasound. Secondly, the PCI was scored on ultrasound by two independent observers and is compared to the PCI value scored ex vivo.

### 3.1. The Outgrowth of Peritoneal Metastases in Rats Can Be Imaged and Followed Using Ultrasound

Ultrasound was applied weekly on all six rats to follow the tumor outgrowth. Tumor lesions were identified by hypoechoic (dark) rounded structures, sometimes surrounded by a hyperechoic (white) halo, and were mostly superficial. Most tumors grew in the cranial part of the abdominal cavity, e.g., around the liver and stomach, but tumor formation also occurred to a lesser extent in the caudal region of the abdominal cavity.

**Figure 4:**
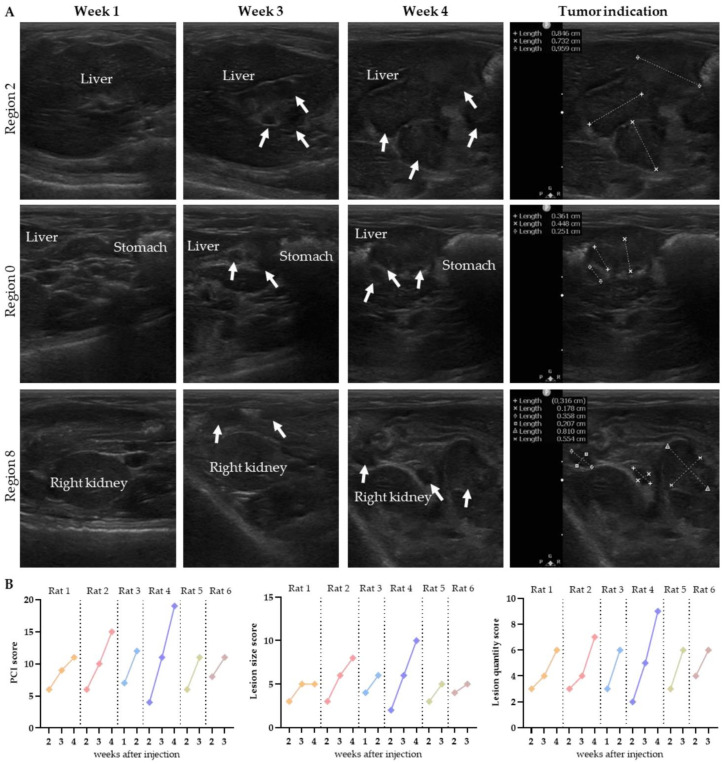
The tumor growth over time is presented for three regions in rat 4 (**A**). Tumor lesions are indicated with white arrows, and organs are named. The progression in total PCI, lesion size and lesion quantity over time was scored on ultrasound by observer 1 (**B**).

In the second week after injection, the presence of tumors was observed adjacent to the liver and stomach, in addition to superficial tumors on the omentum in the middle part of the abdomen. In Figure 4A, the tumor outgrowth over time is presented for rat 4. In Supplementary Figure S2, the outgrowth is presented for other rats.

In most animals, the tumors reached a size of 4–6 mm within 3–4 weeks. Rat 3 was the exception, with much faster tumor outgrowth: the tumors reached a size of 3–8 mm within 2 weeks after injection, and the location of the tumors was limited to the caudal part of the abdomen.

The graphs in Figure 4B present the total PCI, size and quantity of the lesions predicted weekly on ultrasound by observer 1. All scores increased weekly, except for the lesion size score of rat 1 in week 4. In this animal, the size of the tumors in region 2 and 8 increased within the range of the PCI scoring system (4–8 mm), resulting in the same lesion size score. The extent of disease was scored 2 weeks after injection, except for rat 3, in which tumor growth was already observed in the first week after injection. Numerical details are presented in Supplementary Figure S3.

### 3.2. The Predicted PCI on Ultrasound Is Comparable with the Extent of Disease Ex Vivo

**Figure 5:**
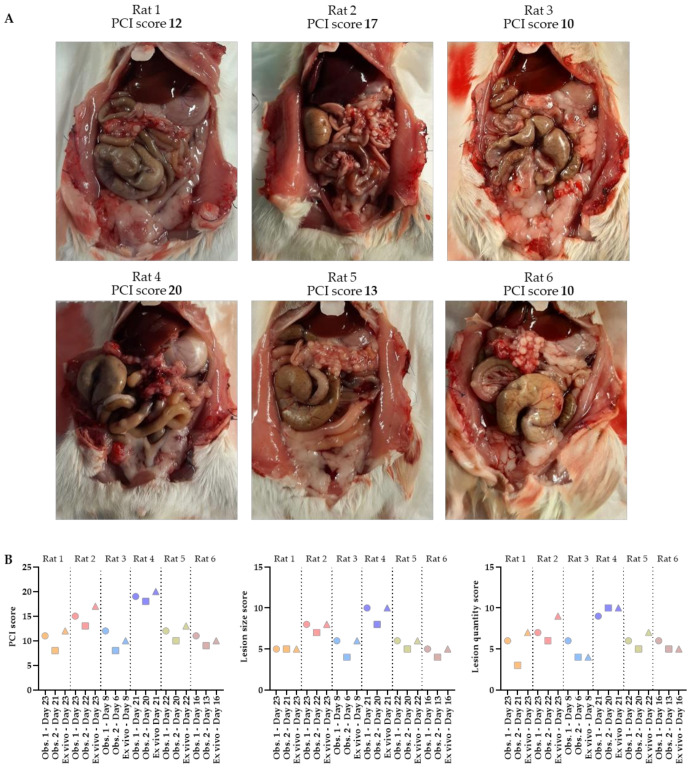
Abdomens of the six rats with corresponding ex vivo PCI scores (**A**). The total PCI, lesion size and lesion quantity scores predicted on ultrasound by 2 independent observers, as well as the ex vivo scoring, are shown in these graphs (**B**). PCI scoring was performed by one of the two observers when tumors reached the size of 4–6 mm, followed by the second ultrasound scoring within 3 days after the animal was sacrificed in order to score the PCI ex vivo.

The total PCI of rats 1–6 was 12, 17, 10, 20, 13 and 10, respectively. The tumor lesions were mainly found in the cranial part of the abdomen, occurring underneath the liver and around the stomach. Specifically, they occurred at the liver hilum, lesser gastric curvature, perisplenic, and on the omentum. In Figure 5A, pictures of the abdomen are presented in which the tumors grew, especially on the omentum. A large number of very small (1–4 mm) tumor lesions were found on the omentum in all six rats.

Before scoring the extent of disease ex vivo, the PCI score was predicted in vivo using ultrasound by two independent observers. Substantial concordance between observer 1 and 2 for scoring both the lesion size and the quantity was found (κ = 0.739 and κ = 0.807, respectively). The total PCI score predicted on ultrasound by observer 1 was slightly higher compared to the scoring of observer 2, with small differences in a range of 1–3 only. In five out of six rats, tumor lesions were scored in the same regions by both observers, which corresponded with the ex vivo results. The only exception was observed in rat 3, in which no tumor lesions were identified in region 6 by observer 2, where tumor lesions were observed by observer 1 and ex vivo. In Table 1, the scoring details are presented.

The graphs representing the total PCI, lesion size and lesion quantity scores show that all predicted measurements are very accurate (Figure 5B). Observer 2 scored the PCI on ultrasound first, followed by the scoring of observer 1 within 3 days. All animals were sacrificed immediately after the last ultrasound, followed by ex vivo PCI scoring performed by observer 1.

In most cases, the total PCI, lesion size and quantity score was initially scored slightly lower by observer 2 than by observer 1 or ex vivo. In rats 1–5, observer 1 predicted the total PCI the best, but in rat 6, both observers only had differences of one compared to the ex vivo PCI.

### 3.3. Comparing Ultrasound Pictures with the Ex Vivo Tumor Load

**Figure 6:**
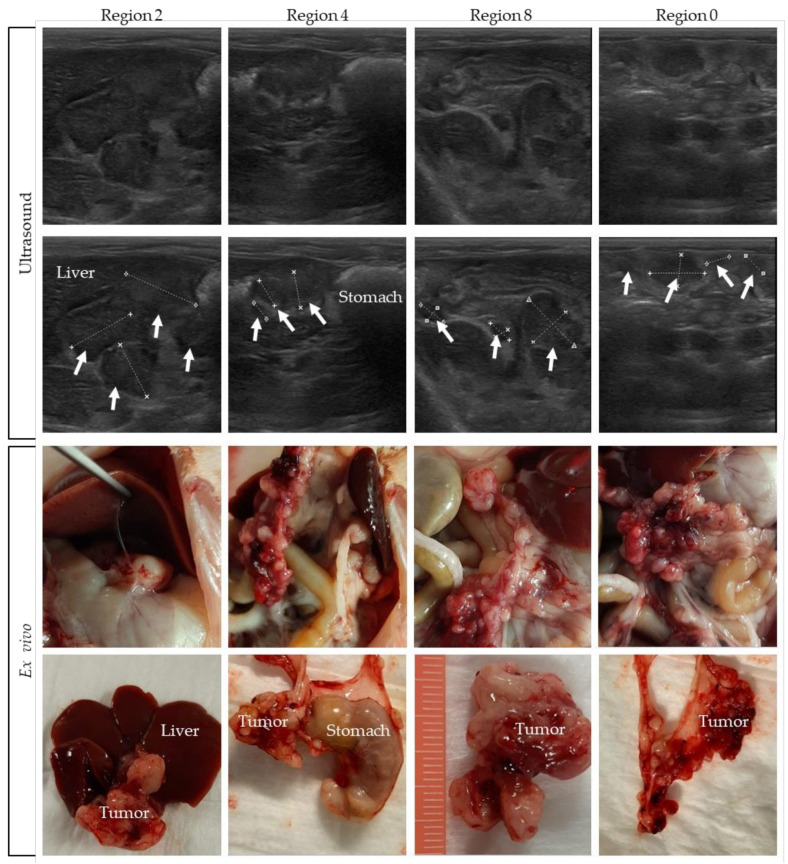
Tumors on ultrasound compared to the ex vivo tumor load. Tumor lesions are indicated with white arrows, and organs are named.

Pictures were taken and tumor size was measured during the dissection of the rats. In Figure 6, pictures of rat 4 are compared with the corresponding ultrasound indication of four different regions. In the upper panel of the figure, ultrasound pictures are shown, with the tumors indicated in the second row of pictures. The ex vivo pictures are presented in the lower panel. In Supplementary Figure S4, tumor images on ultrasound are compared to the ex vivo tumor load of the other rats.

Comparing the ex vivo tumor size of all individual tumors in a specific region with ultrasound-based size measurements is very difficult, especially for the many smaller lesions. On ultrasound only, part of the tumor load is visible, which makes it difficult to compare it with the exact same region ex vivo. Nevertheless, these figures show that, overall, a very accurate tumor assessment can be performed on ultrasound.

## 4. Discussion

A representative orthotopic animal model is important when using a preclinical research model to study peritoneal dissemination and the effectiveness of intraperitoneal chemotherapy [12]. A major challenge for the optimal use of orthotopic animal models is non-invasive identification of tumor nodules and monitoring tumor outgrowth over time. To the best of our knowledge, the present study is the first to demonstrate the use of ultrasound to monitor tumor outgrowth and the extent of disease in orthotopic PM rat models.

In all six WAG/Rij rats, intraperitoneal injection with CC-531 cells resulted in tumor lesions spread throughout the abdominal cavity. By performing weekly ultrasound, we were able to identify tumor nodules and determine the PCI scores. Repeat imaging revealed increasing PCI values every week (Figure 5). Although the total PCI scoring was slightly lower on ultrasound, imaging the location, size and quantity was successful and reproducible on ultrasound. These results also show that even without extensive ultrasound experience, the extent of disease can be predicted very well using ultrasound. These results confirm that ultrasound can be used as a non-invasive imaging modality for the non-invasive detection and growth monitoring of tumor lesions, over time, and to determine tumor spread in the abdominal cavity of small animals.

We strongly recommend following the step-by-step procedure presented in Figure 2 for an optimal and complete imaging of the peritoneal cavity, which increases the chance of identifying all lesions in this area. To avoid observer variability, one observer should perform the ultrasound measurements during the entire experiment. We would recommend having the measurements performed by two independent observers to obtain the most reliable results. In this way, the optimal treatment moment can be determined based on the actual extent of disease.

When using another cell line than CC-531 cells to induce PM, we recommend testing the efficacy of ultrasound first for the specific lesion size and patterns of the other cell line. Besides differences in lesion size, quantity or location, the tumors can be hypo- or hyper-echoic and therefore the accuracy should be assessed before starting experiments.

Overall, the PCI scoring by observer 1 was slightly higher compared to that by observer 2 (Figure 6). The concordance between the observers regarding identified regions with tumor nodules was very high, with the only discrepancy being in region 6 of rat 3 (Table 1). There are several possible explanations for this discrepancy: observer 2 did not score any small tumors, as there were no tumors present at the time observer 2 examined rat 3; observer 2 did not find any tumor present in region 6 as there was air disturbing the image; or observer 2 performed a less accurate ultrasound analysis for this specific area. To prevent this last issue, it is strongly advised to use a standard procedure to always image the entire abdominal cavity, to prevent missing a region.

The small differences of the lesion size scoring between both observers can be explained by the time between their ultrasound sessions. In theory, a lesion size of 4 mm was measured in a certain region by observer 2, resulting in an LS score of 1. Within 3 days, the size of the lesion can easily increase with 1 mm, resulting in a lesion size of 5 mm, resulting in an LS score of 2 scored by observer 1, both on ultrasound and ex vivo. Small size differences, but also one extra lesion in a certain area, can result in a different PCI score.

Measurements of the last animals (rat 5 and 6) differ only with a score of 1 between both observers, suggesting that there may be a fairly rapid learning curve. Although the differences were small, our data do show that a minimal level of experience with ultrasound is preferable, and that following the guidelines and practice is sufficient to accurately detect tumor formation and score the extent of disease.

The drawbacks of bioluminescence imaging in the detection of PM in small animal models can be overcome by using ultrasound. When multiple tumors are present in the peritoneum, a single large positive stain will appear when using bioluminescence imaging, while small tumor lesions can be identified separately on ultrasound. Although ultrasound images are two-dimensional, it is feasible to image tumors from different sides, resulting in more accurate PCI scoring.

However, some limitations when using ultrasound should be taken into account. Sometimes defining the tumor load proves to be difficult, especially when the stomach and intestine are filled. To solve this problem, it is recommended to image animals after 3–7 h of food deprivation [30,31], depending on the strain and size of the animals.

A more exact comparison of ultrasound- and ex vivo-based tumor lesion size measurements is very difficult. In every ultrasound probe position, a minimal part of the animal, and therefore also of the tumor load, is visible. An indication of the tumor location in a certain area can be made, but comparing that exact same tumor to the ex vivo tumor is very difficult, especially for small lesions.

The use of modern ultrasound techniques, e.g., elastography or contrast-enhanced, can be considered to improve the quality of the current ultrasound. Elastography images the elastic properties of soft tissues, providing useful information about pathological characteristics. Using this technique, tumors can be identified, but also the efficacy of therapeutic agents can be monitored [32]. The addition of contrast-enhanced ultrasound improves the visualization of blood vessels and organs, which in turn enables the detection of tumor lesions [33,34]. This imaging modality requires an additional injection before ultrasound, which limits the feasibility of repeated imaging in animal models. The major disadvantages of modern ultrasound techniques are the high costs and the lack of experiences for the application on peritoneal metastasis animal models.

However, we were still able to achieve an adequate overall assessment, as illustrated in Figure 6, where we showed that a tumor location on ultrasound can be quite accurately compared with the ex vivo result. The correct overall assessment of the extent of PM for scoring the PCI can be used to plan the optimal timing of performing experiments in an orthotopic PM animal model precisely. The latter is important to guarantee that the PM and PCI of different animals within the cohort represent the same disease stage at the intervention, and that results of the intervention can be evaluated and compared for different studies.

## 5. Conclusions

Ultrasound can be used as a non-invasive tool for the imaging and scoring of PM in small animal models. To the best of our knowledge, our data indicate for the first time that metastatic spread through the peritoneum can be detected and the extent of disease can be scored accurately in small animals using ultrasound, even for users with minimal ultrasound experiences. Monitoring the tumor outgrowth reduces the number of animals needed, thereby increasing homogeneity between the animals regarding tumor sizes and improving data reproducibility.

## Figures and Tables

**Table 1:** Numerical details on the PCI scoring.

	**Rat 1**						**Rat 2**						**Rat 3**					
**Regions**	**Lesion Size**			**Lesion Quantity**			**Lesion Size**			**Lesion Quantity**			**Lesion Size**			**Lesion Quantity**		
	Obs. 1	Obs.2	Ex vivo	Obs. 1	Obs.2	Ex vivo	Obs. 1	Obs.2	Ex vivo	Obs. 1	Obs.2	Ex vivo	Obs. 1	Obs.2	Ex vivo	Obs. 1	Obs.2	Ex vivo
**0 Central**	1	2	2	2	1	3	2	2	2	2	3	3	1	1	1	1	1	1
**1 Right Upper**																		
**2 Epigastrium**	2	2	1	2	1	2	2	2	2	2	1	2						
**3 Left Upper**							··											
**4 Left Flank**							··2	2	2	··2	1	··3						
**5 Left Lower**																		
**6 Pelvis**													2··		2	1··		1
**7 Right Lower**													1	1	1	2	1	1
**8 Right Flank**	2	1	2	2	1	2	2	1	2	1	1	1	2	2	2	2	2	1
**Subtotal**	5	5	5	6	3	7	8	7	8	7	6	9	6	4	6	6	4	4
**Total PCI**	**Obs. 1**		**Obs. 2**		**Ex vivo**		**Obs. 1**		**Obs. 2**		**Ex vivo**		**Obs. 1**		**Obs. 2**		**Ex vivo**	
	**11**		**8**		**12**		**15**		**13**		**17**		**12**		**8**		**10**	
	**Rat 4**						**Rat 5**						**Rat 6**					
**Regions**	**Lesion Size**			**Lesion Quantity**			**Lesion Size**			**Lesion Quantity**			**Lesion Size**			**Lesion Quantity**		
	Obs. 1	Obs.2	Ex vivo	Obs. 1	Obs.2	Ex vivo	Obs. 1	Obs.2	Ex vivo	Obs. 1	Obs.2	Ex vivo	Obs. 1	Obs.2	Ex vivo	Obs. 1	Obs.2	Ex vivo
**0 Central**	3	2	3	3	3	3	1	2	2	2	2	3	2	2	2	2	2	2
**1 Right Upper**																		
**2 Epigastrium**	3	2	3	2	2	3	2	2	2	2	2	2	1	1	1	2	2	2
**3 Left Upper**																		
**4 Left Flank**	2	2	2	2	2	2	1	1	1	1	1	1						
**5 Left Lower**																		
**6 Pelvis**																		
**7 Right Lower**																		
**8 Right Flank**	2	2	2	2	3	2	1		1	1		1	2	1	2	2	1	1
**Subtotal**	10	8	10	9	10	10	5	5	6	6	5	7	5	4	5	6	5	5
**Total PCI**	**Obs. 1**		**Obs. 2**		**Ex vivo**		**Obs. 1**		**Obs. 2**		**Ex vivo**		**Obs. 1**		**Obs. 2**		**Ex vivo**	
	**19**		**18**		**20**		**11**		**10**		**13**		**11**		**9**		**10**	

## Data Availability

Not applicable.

## Supplementary Materials

The following supporting information can be downloaded at: https://www.mdpi.com/article/10.3390/biomedicines10071610/s1, Figure S1: Ultrasound settings; Figure S2: The tumor growth over time is presented for three regions in rats 1, 2, 3, 5 and 6; Figure S3: Numerical details on the total PCI, lesion size and lesion quantity scored on ultrasound by observer 1 over time; Figure S4: Tumors on ultrasound compared to the ex vivo tumor load for rats 1, 2, 3, 5 and 6.
