# Supplementary material for: Non-Invasive Imaging and Scoring of Peritoneal Metastases in Small Preclinical Animal Models Using Ultrasound: A Preliminary Trial
Source: Biomedicines. 2022 Jul 6;10(7):1610. doi: 10.3390/biomedicines10071610 (PMC9313051; doi:10.3390/biomedicines10071610)

Supplementary Figure S1. Ultrasound settings

**Ultrasound parameters**	**Setting**
System	NZE1700 CX50
Transducer	L15-7io
Depth	2.5 cm
Frequency	42 Hz
Mode	2D, Res
Focal zone	1-2
Gain (Gn)	90
Compress (C)	66
Decibels	-0.3 dB
Mechanical Index (MI)	0.5
Thermal Index Soft Tissue (TIS)	0.1

Supplementary Figure S2 . The tumor growth over time is presented for three regions in rat 1,2,3,5 and 6

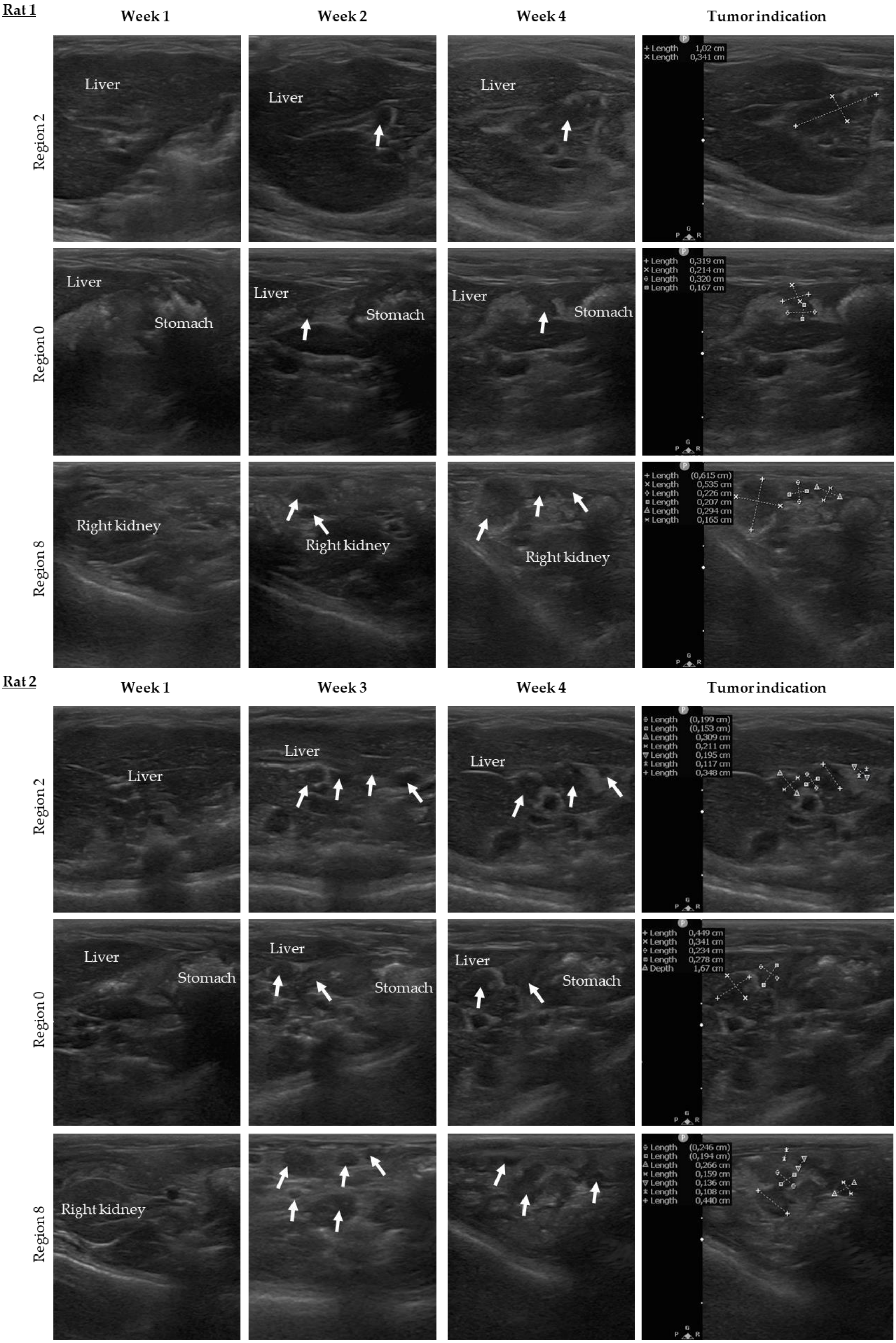

Supplementary Figure S2. The tumor growth over time is presented for three regions in rat 1,2,3,5 and 6

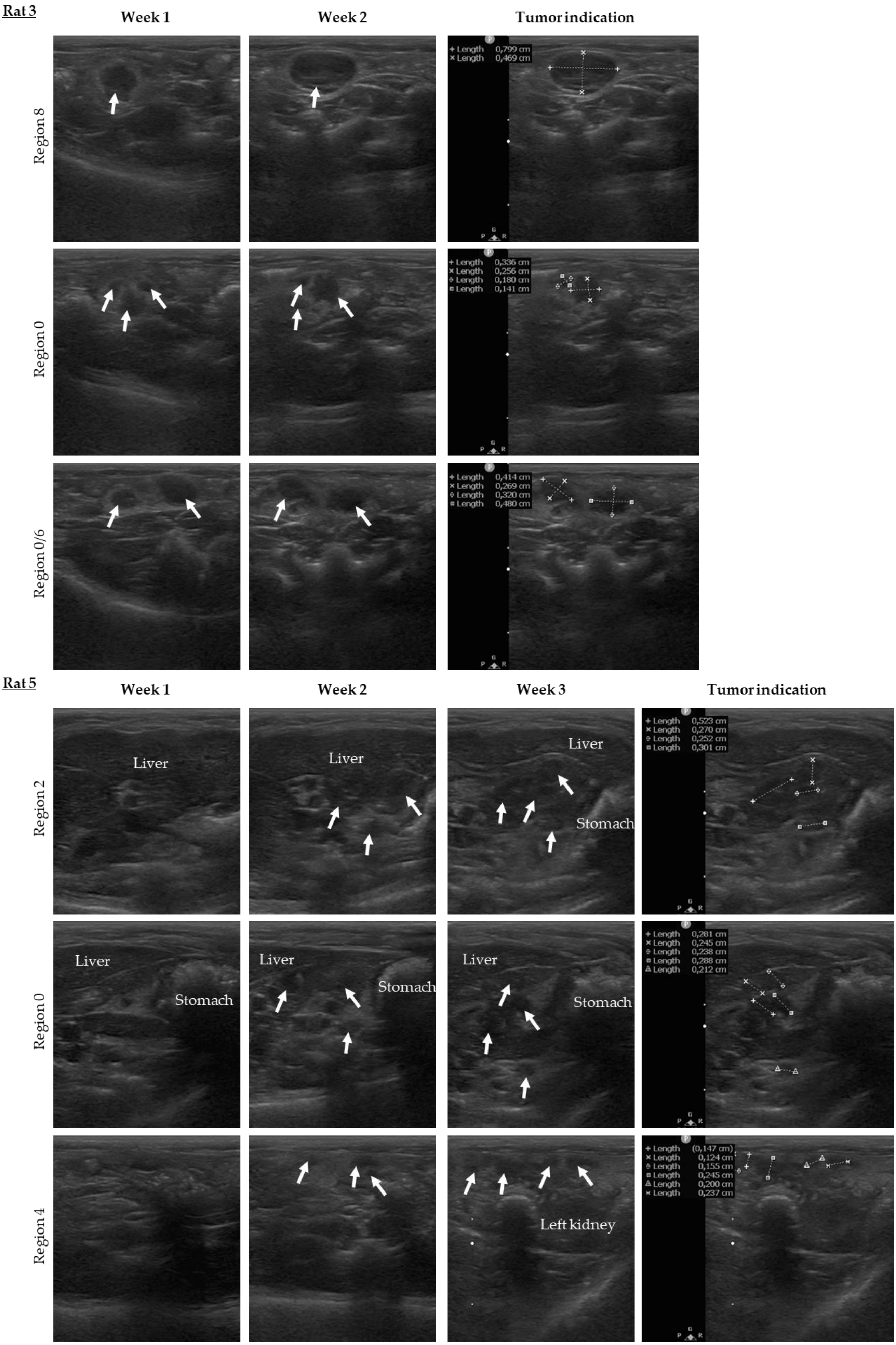

Supplementary Figure S2. The tumor growth over time is presented for three regions in rat 1,2,3,5 and 6.

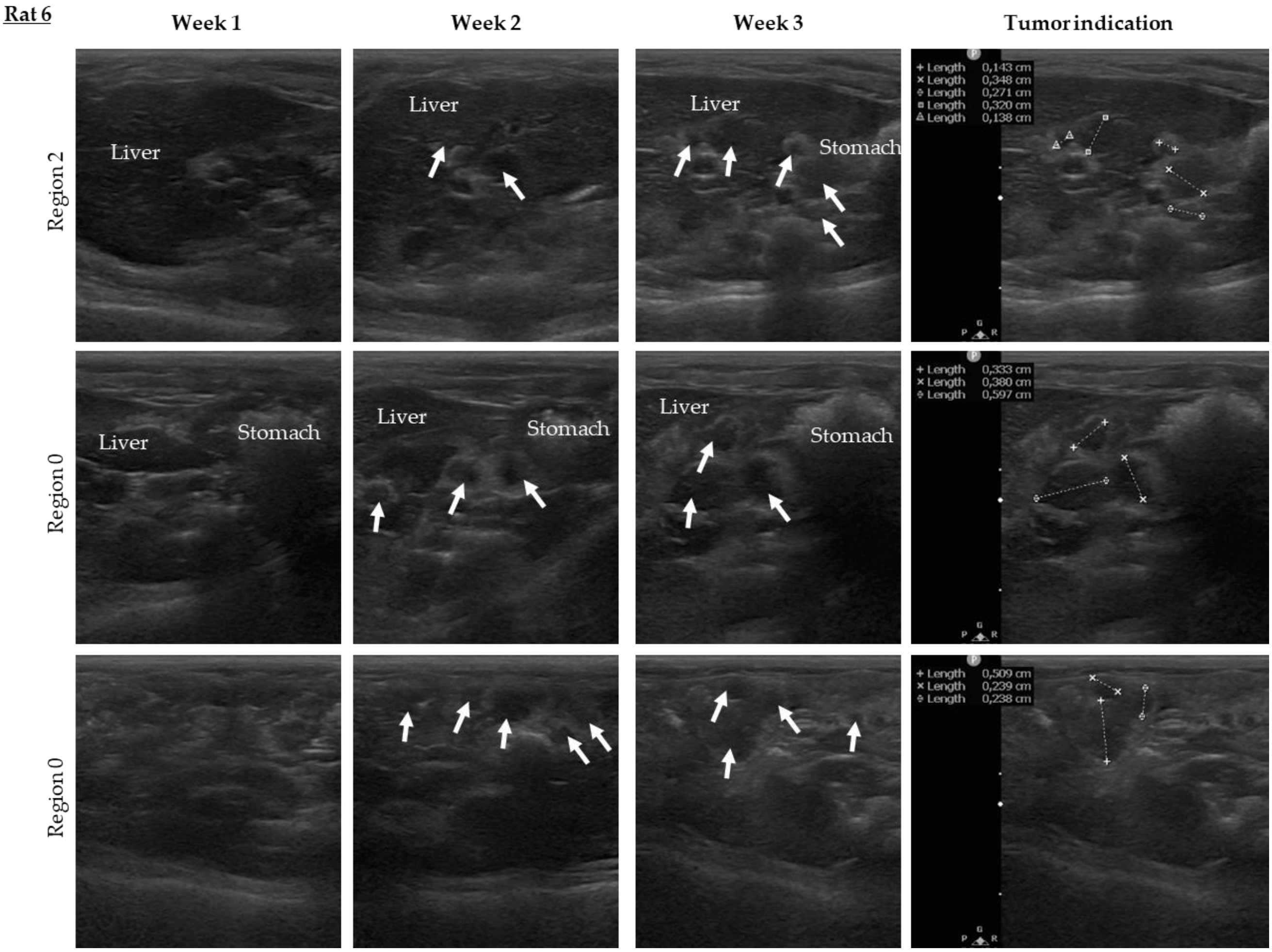

Supplementary Figure S3. Numerical details on the total PCI, lesion size and lesion quantity scored on ultrasound by observer 1 over time.

	Rat 1						Rat 2						Rat 3			
**Regions**	**Lesion Size**			**Lesion Quantity**			**Lesion Size**			**Lesion Quantity**			**Lesion Size**		**Lesion Quantity**	
	Week 2	Week 3	Week 4	Week 2	Week 3	Week 4	Week 2	Week 3	Week 4	Week 2	Week 3	Week 4	Week 2	Week 3	Week 2	Week 3
**0 Central**	1	1	1	1	1	2	1	2	2	1	1	2		1		1
**1 Right Upper**																
**2 Epigastrium**	1	2	2	1	1	2	1	1	2	1	1	2				
**3 Left Upper**																
**4 Left Flank**								1	2		1	2				
**5 Left Lower**																
**6 Pelvis**													1	2	1	1
**7 Right Lower**													1	1	1	2
**8 Right Flank**	1	2	2	1	2	2	1	2	2	1	1	1	2	2	1	2
**Subtotal**	**3**	**5**	**5**	**3**	**4**	**6**	**3**	**6**	**8**	**3**	**4**	**7**	**4**	**6**	3	6

	Rat 1			Rat 2			Rat 3	
	Week 2	Week 3	Week 4	Week 2	Week 3	Week 4	Week 1	Week 2
**Total PCI**	**6**	**9**	**11**	**6**	**10**	**15**	**7**	**12**

	Rat 4						Rat 5				Rat 6			
**Regions**	**Lesion Size**			**Lesion Quantity**			**Lesion Size**		**Lesion Quantity**		**Lesion Size**		**Lesion Quantity**	
	Week 2	Week 3	Week 4	Week 2	Week 3	Week 4	Week 2	Week 3	Week 2	Week 3	Week 2	Week 3	Week 2	Week 3
**0 Central**		1	3		2	3	1	1	1	2	1	2	2	2
**1 Right Upper**														
**2 Epigastrium**	1	2	3	1	1	2	1	2	1	2	1	1	1	2
**3 Left Upper**														
**4 Left Flank**		1	2		1	2	1	1	1	1				
**5 Left Lower**														
**6 Pelvis**														
**7 Right Lower**														
**8 Right Flank**	1	2	2	1	1	2		1		1	2	2	1	2
**Subtotal**	**2**	**6**	**10**	**2**	**5**	**9**	**3**	**5**	**3**	**6**	**4**	**5**	**4**	**6**

	Rat 4			Rat 5		Rat 6	
	Week 2	Week 3	Week 4	Week 2	Week 3	Week 2	Week 3
**Total PCI**	**4**	**11**	**19**	**6**	**11**	**8**	**11**

Supplementary Figure S4. Tumors on ultrasound compared to the *ex vivo* tumor load for rat 1, 2, 3, 5 and 6

Rat 1 – PCI score of 12

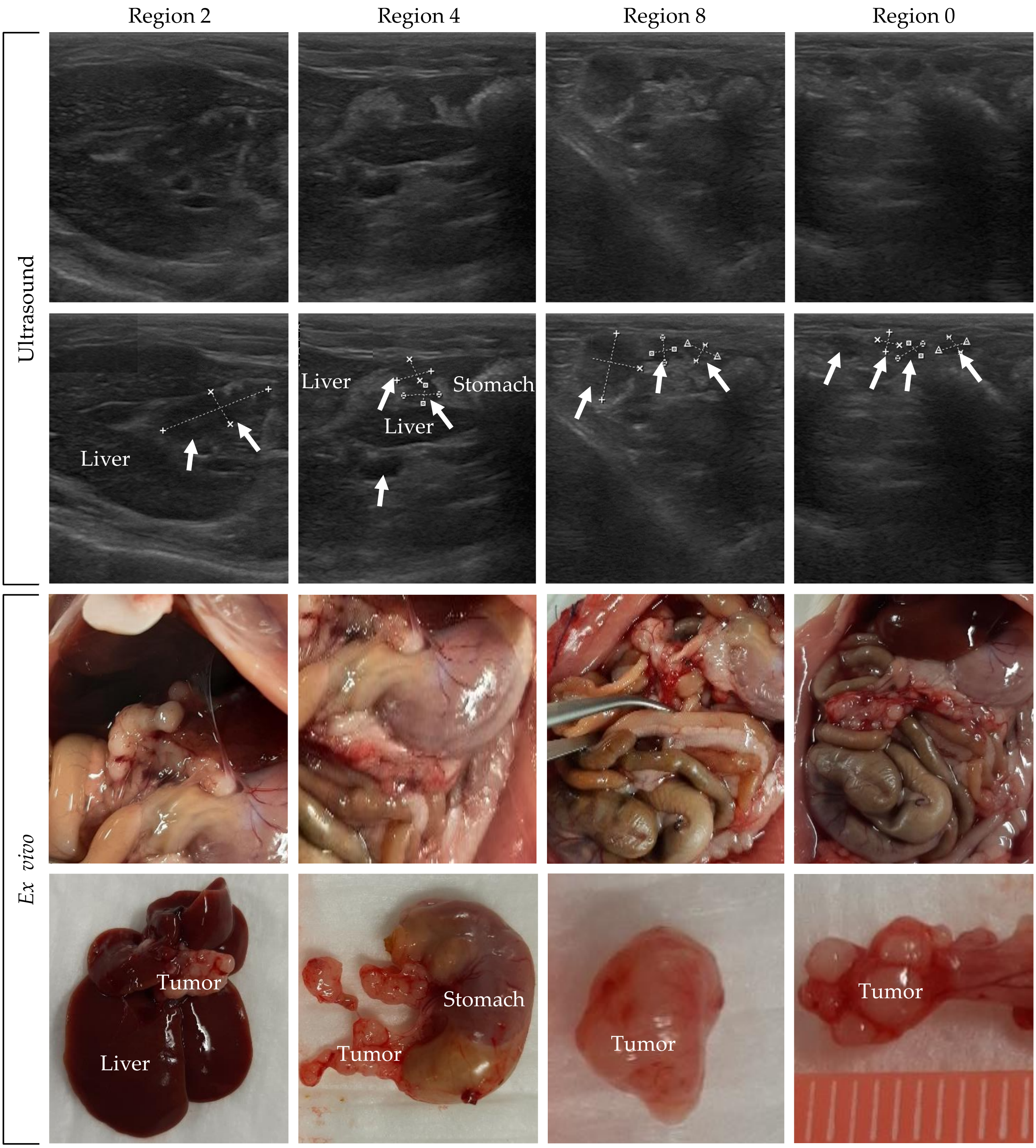

Supplementary Figure S4. Tumors on ultrasound compared to the *ex vivo* tumor load for rat 1, 2, 3,

5 and 6

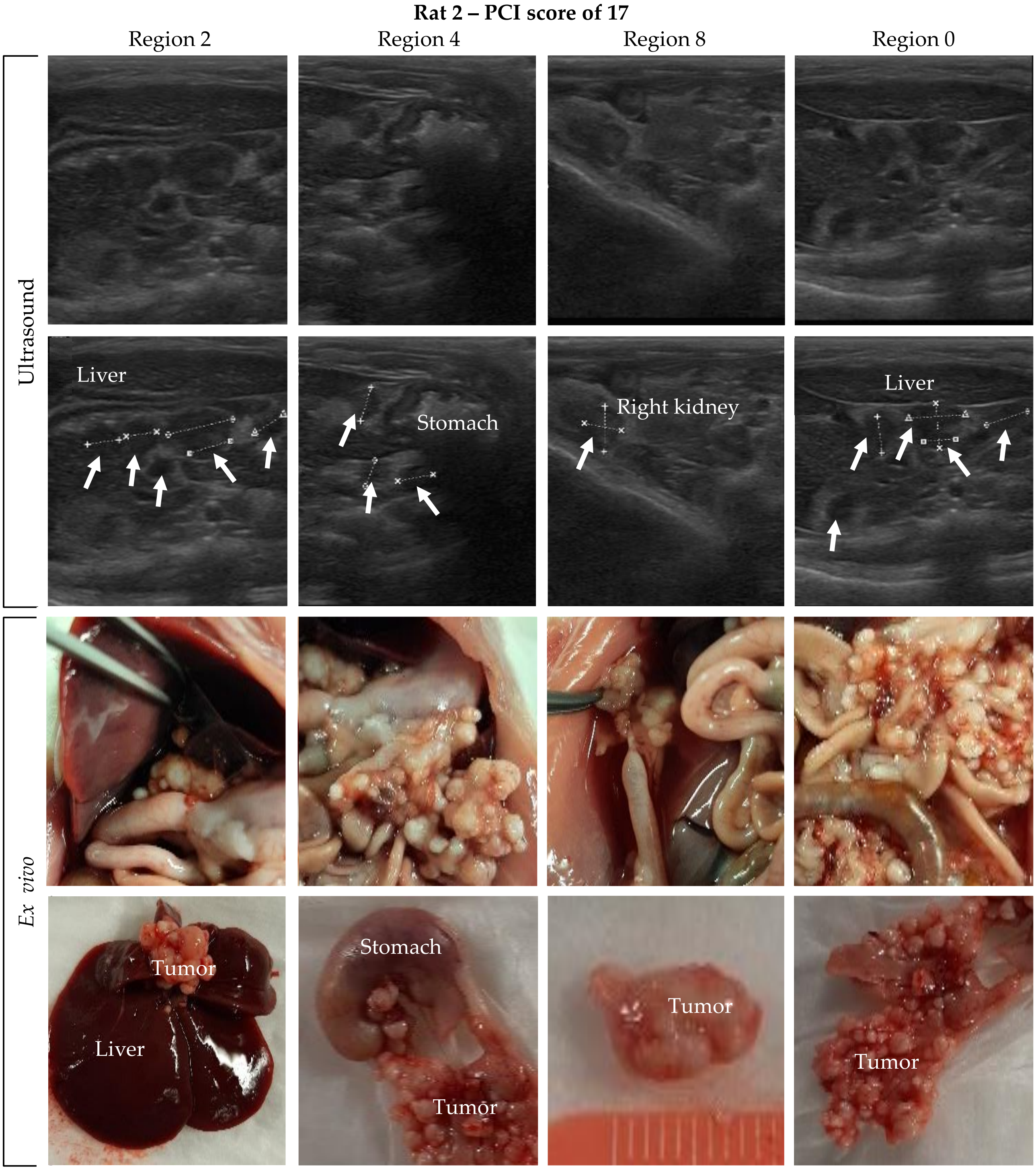

Supplementary Figure S4. Tumors on ultrasound compared to the *ex vivo* tumor load for rat 1, 2, 3,

5 and 6

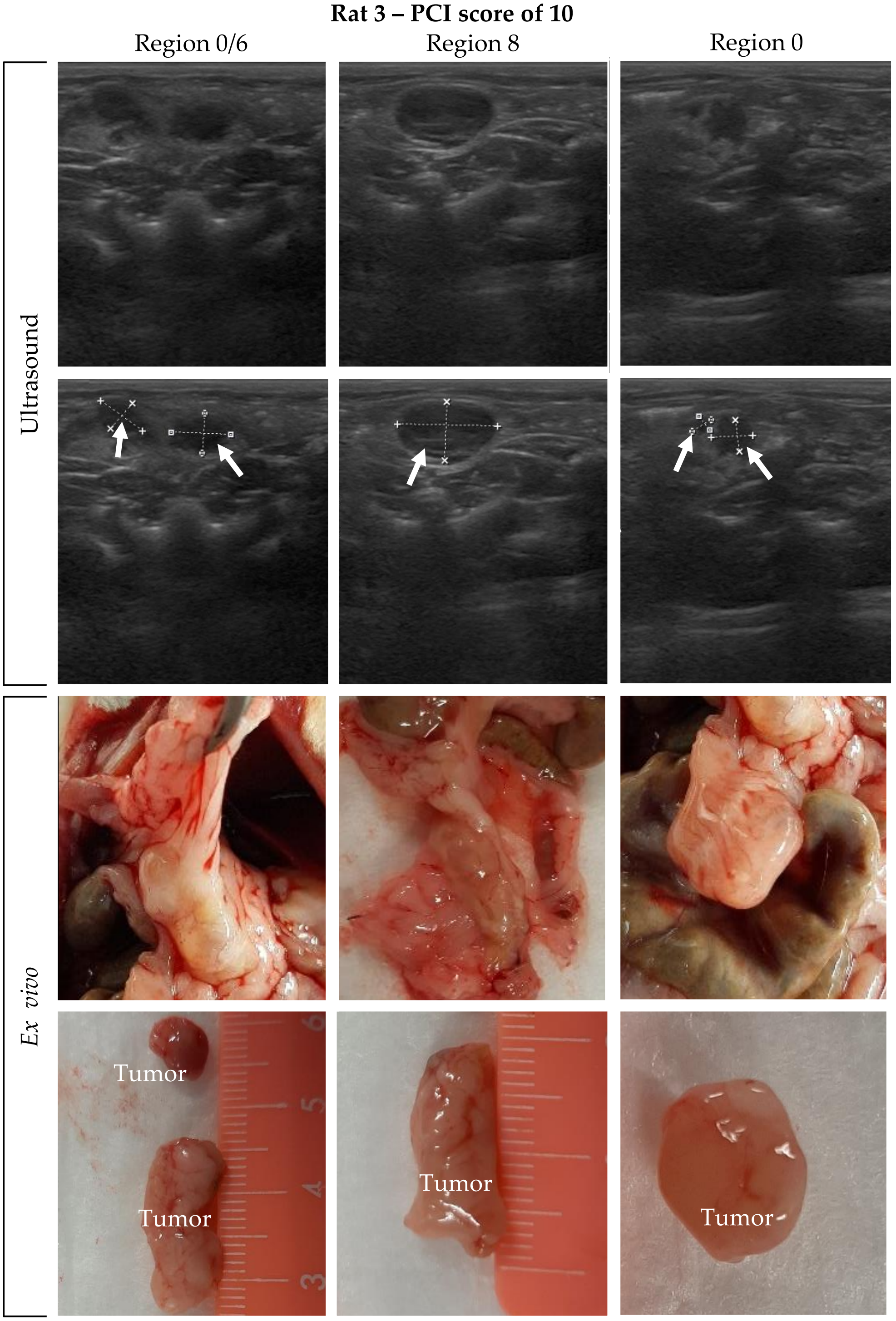

Supplementary Figure S4. Tumors on ultrasound compared to the *ex vivo* tumor load for rat 1, 2, 3,

5 and 6

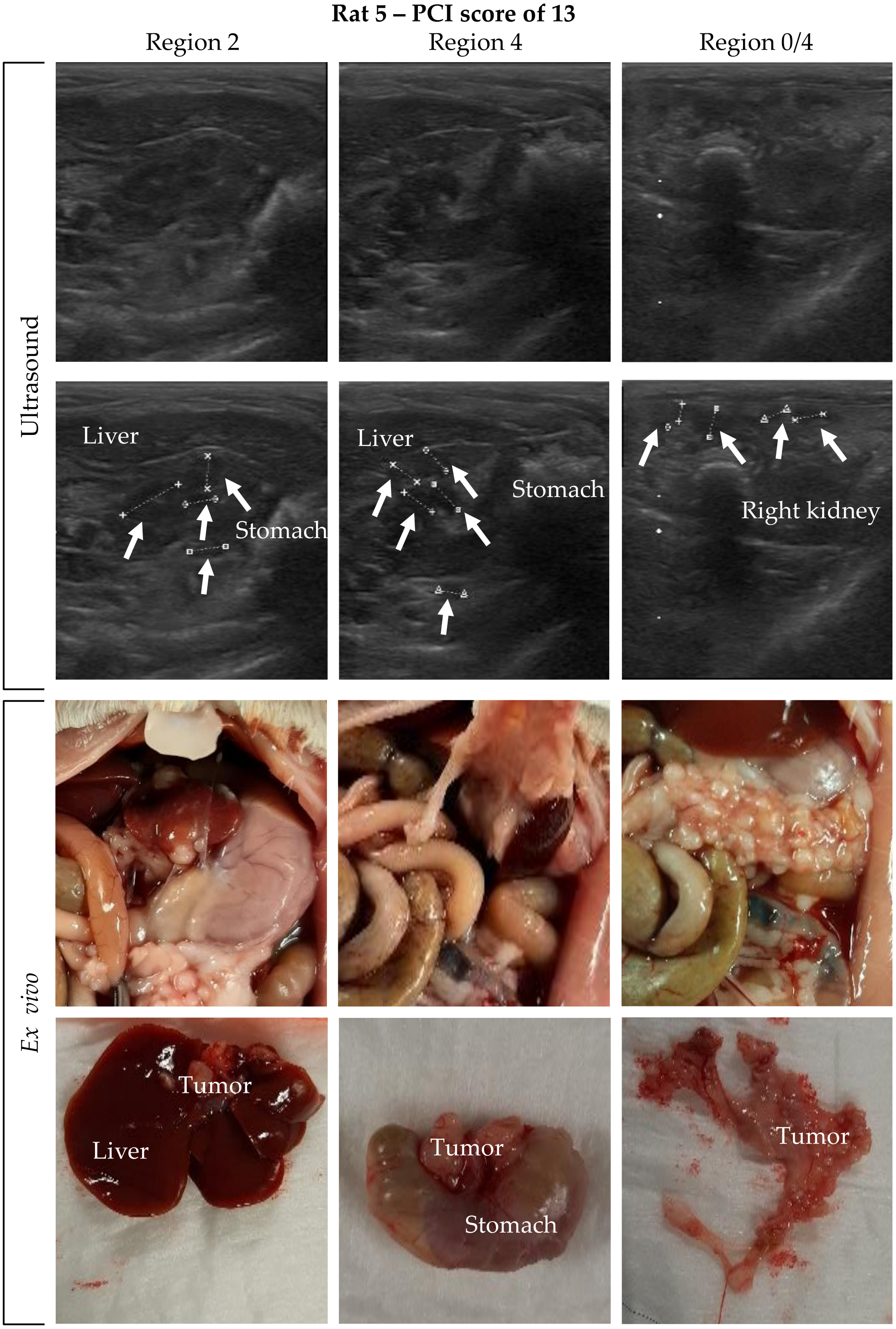

Supplementary Figure S4. Tumors on ultrasound compared to the *ex vivo* tumor load for rat 1, 2, 3, 5 and 6

Rat 6 – PCI score of 10

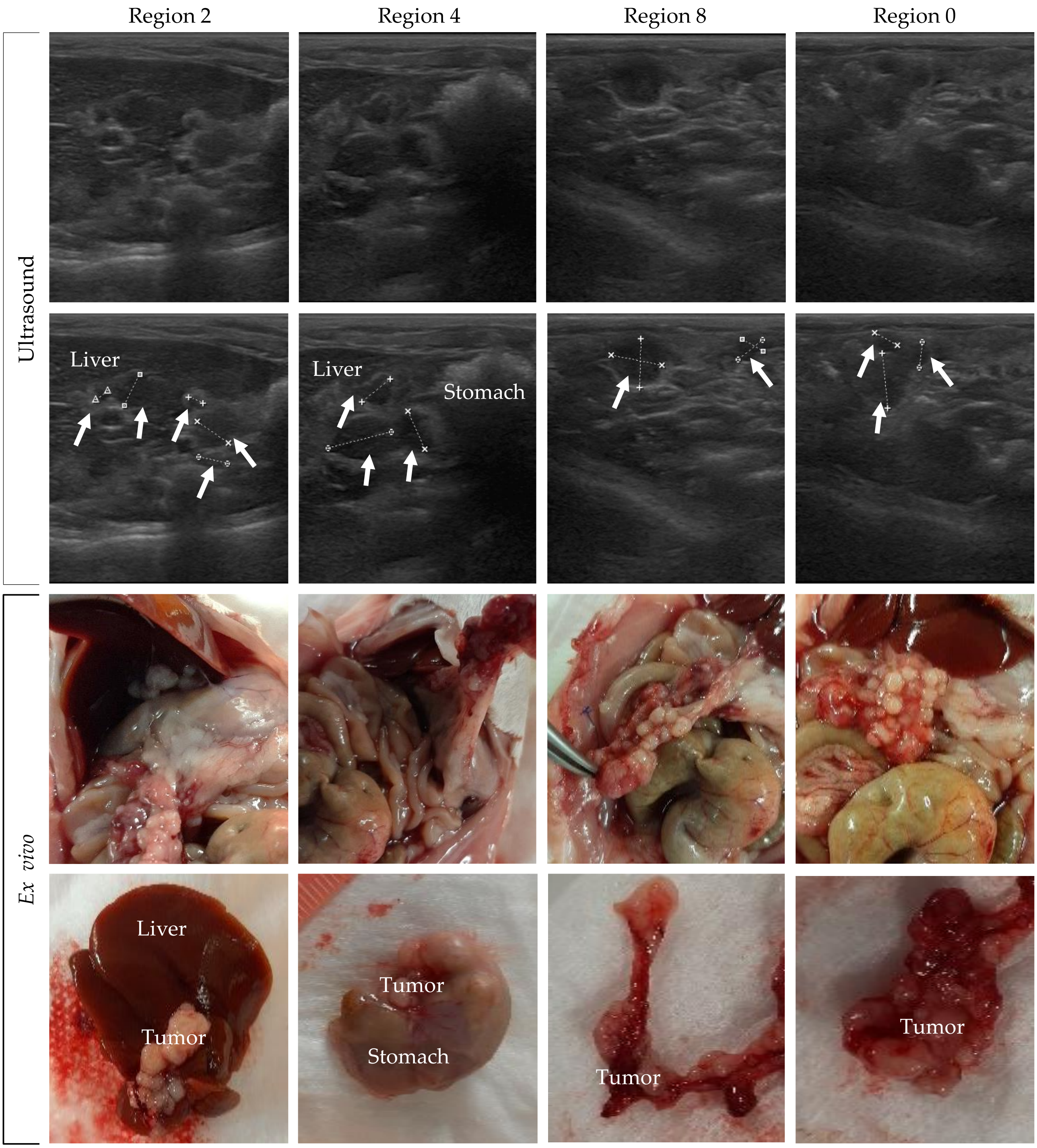

Supplement: Supplementary file 1 [file biomedicines-10-01610-s001.zip › biomedicines-1692163-supplementary.pdf]
